# Assessment of Immune Status Using Inexpensive Cytokines: A Literature Review and Learning Approaches

**DOI:** 10.3390/s22249785

**Published:** 2022-12-13

**Authors:** Noor Jamaludeen, Juliane Lehmann, Christian Beyer, Katrin Vogel, Mandy Pierau, Monika Brunner-Weinzierl, Myra Spiliopoulou

**Affiliations:** 1 Knowledge Management & Discovery Lab, Otto-von-Guericke University, 39106 Magdeburg, Germany; 2 Department of Experimental Pediatrics, University Hospital, Otto-von-Guericke University, 39120 Magdeburg, Germany

**Keywords:** point of care, at home, cytokine, regression, decision tree, random forest, PRISMA, literature review

## Abstract

The emergence of point-of-care (POC) testing has lately been promoted to deliver rapid, reliable medical tests in critical life-threatening situations, especially in resource-limited settings. Recently, POC tests have witnessed further advances due to the technological revolution in smartphones. Smartphones are integrated as reliable readers to the POC results to improve their quantitative detection. This has enabled the use of more complex medical tests by the patient him/herself at home without the need for professional staff and sophisticated equipment. Cytokines, the important immune system biomarkers, are still measured today using the time-consuming Enzyme-Linked Immunosorbent Assay (ELISA), which can only be performed in specially equipped laboratories. Therefore, in this study, we investigate the current development of POC technologies suitable for the home testing of cytokines by conducting a PRISMA literature review. Then, we classify the collected technologies as inexpensive and expensive depending on whether the cytokines can be measured easily at home or not. Additionally, we propose a machine learning-based solution to even increase the efficiency of the cytokine measurement by leveraging the cytokines that can be inexpensively measured to predict the values of the expensive ones. In total, we identify 12 POCs for cytokine quantification. We find that Interleukin 1β (IL-1β), Interleukin 3 (IL-3), Interleukin 6 (IL-6), Interleukin 8 (IL-8) and Tumor necrosis factor (TNF) can be measured with inexpensive POC technology, namely at home. We build machine-learning models to predict the values of other expensive cytokines such as Interferon-gamma (IFN-γ), IL-10, IL-2, IL-17A, IL-17F, IL-4 and IL-5 by relying on the identified inexpensive ones in addition to the age of the individual. We evaluate to what extent the built machine learning models can use the inexpensive cytokines to predict the expensive ones on 351 healthy subjects from the public dataset 10k Immunomes. The models for IFN-γ show high results for the coefficient of determination: $R^{2}$ = 0.743. The results for IL-5 and IL-4 are also promising, whereas the predictive model of IL-10 achieves only $R^{2}$ = 0.126. Lastly, the results demonstrate the vital role of TNF and IL-6 in the immune system due to its high importance in the predictions of all the other expensive cytokines.

## 1. Introduction

Point of care (POC) tests also play a significant role in combating the COVID-19 virus, as any person can test him or herself at home for the presence of an infection with the virus within a few minutes. As the above examples show, POC tests are used for various purposes, including monitoring therapy, managing a disease or maintaining the health of a patient. Thereby, the patient is given more responsibility but also more control over him or herself.

In developing POC tests, researchers have also recognized the potential of recent technology developments such as smartphones and machine learning (e.g., [1,2]). This is not surprising, as there are 3.9 billion smartphone users worldwide. In Germany alone, there are 62.6 million users, which corresponds to a proportion of 88.8 percent of the total population of the country [3]. Furthermore, Smartphones are capable of quite a lot these days. They have good cameras, many connecting options for peripheral devices, Internet access, various sensors, GPS, the possibility of downloading special software in the form of apps and, best of all, we always have them with us. This makes it also interesting to monitor the status of the immune system in critical health conditions in resource-limited settings or if you are alone at home without the assistance of a physician or other medical staff. Important biomarkers of the immune system are the so-called cytokines, which can provide information on the development and course of diseases and inflammatory processes [4]. The gold standard method for measuring cytokine levels nowadays is still the enzyme-linked immunosorbent assay (ELISA); the biggest downside is that it is very time consuming and therefore not suitable for at-home testing by the patient him/herself or use in critical health conditions where time is crucial [5,6,7]. In contrast, POC tests are rapid and there is no need for specially trained laboratory personnel or facilities [4].

Therefore, it is necessary to identify the state of development of cytokine tests suitable for home usage by the patient him/herself, especially those that require little or already at-home existing equipment—such as a smartphone.

Furthermore, machine learning algorithms have been widely used in the medical domain. A machine learning model is used to predict the levels of inflammatory cytokines in patients with Parkinson’s disease [8]. Wei et al. train a random forest classifier to predict potential cytokine–receptor interactions [9]. Subsequently, we believe that machine learning methods have the potential to enhance the quantification of cytokines even more. In the course of this study, the following research questions are to be answered (RQ_1_–RQ_3_). The distinction between expensive and inexpensive is made based on how easy it is to acquire the cytokines with the technologies identified. In other words, the detection of cytokines is categorized as inexpensive if the technology used to measure the cytokines is mature and uses equipment that is available in most households, such as a smartphone or a sheet of paper. Otherwise, the technology is categorized as expensive.

This study aims at answering three research questions:

- RQ_1_: What recent technologies developed for the rapid determination of cytokines are suitable for at-home testing?
- RQ_2_: Which technologies suitable for at-home testing can be used to acquire cytokines inexpensively and which expensively and which cytokines do these technologies measure?
- RQ_3_: To what extent can cytokines measured by inexpensive technologies be used to predict levels of cytokines that can currently be tested only by expensive technologies based on the public dataset 10k Immunomes?

In this study, our main contributions can be described as follows:

1. We use the PRISMA method to collect POC technologies that can be used for at-home testing with a focus on methods that leverage smartphones for cytokine quantification to answer RQ_1_.
2. To answer RQ_2_, we group the retrieved POC technologies into expensive and inexpensive cytokines based on the equipment and the personnel required to conduct the test.
3. We answer RQ_3_ by building machine learning models that take as input the inexpensive cytokines and predict each of the expensive ones. Then, we evaluate the performance of the models to validate the potential of delivering reliable predictions for each of the expensive cytokines. Thus, the quantification of expensive cytokines can be carried out without the need for any expensive test.

This work is structured as follows: in Section 2, we discuss related work. We provide a detailed description of our method to answer the research questions RQ_1_ and RQ_2_ in Section 3. We evaluate it and discuss the results in Section 4. In Section 5, we describe the public dataset that we use for evaluation. Section 6 presents the regression analysis of healthy subjects of a public dataset to answer the research questions RQ_3_. The regression analysis results are presented in Section 7. Finally, a summary of the study and an outlook including achievements is discussed in Section 8.

## 2. Related Work

In 2021, we published a review [10] on the subject area of the research questions. We looked into immunoassays that can measure the immune status at home or the point of care using cytokines in the blood [10]. As a result, the research group found 15 assays for the rapid detection of cytokines with a maximum assay time of 60 min and sample types such as unprocessed blood or processed blood samples (e.g., plasma or serum) [10]. Most of the found assays can measure a single cytokine only and are suitable for application at the point of care [10]. Very few found technologies “could be with some slight development mature enough for home-usage” [10] (p. 28). Furthermore, Jamaludeen et al. investigated health questionnaires that collect additional information (e.g., chronic diseases, psychological issues) and thus have the potential to support the quality of the analysis of immune status by the found assays [10]. We also suggested a machine learning approach to derive automatically from the literature a definition for “immune fitness” and a suggestion for characteristics of technologies or assays suitable for at-home usage [10].

Liu et al. summarized on account of the COVID-19 pandemic in their 2021 review [2] the most recent technologies for cytokine detection at the POC and presented an overview of diseases resulting in increased or imbalanced levels of cytokines (e.g., sepsis, COVID-19), including the names of the cytokines, the threshold values of the selected cytokines and the sample types (e.g., whole blood, plasma, serum) used [2]. Furthermore, the research group discussed the challenges and perspectives of POC technologies concerning the ASSURED criteria [2]. The challenges the research group discovered are, for instance, the low detection sensitivity of POC technologies, as well as the “limited multiplexing ability (only with the capability of detecting <10 cytokines at the moment) because of lots of challenges such as limited signal readout, technical variation between different labs, cross-reactivity effects” [2] (p. 13). Liu et al. pointed out the potential of POC technologies in combination with smartphones, for example, to read the results of the measured cytokine levels directly from the display and the benefits of the application of machine learning [2].

Hernández-Neuta et al. investigated in their 2019 review [1] smartphone-based diagnostic and its contributing technologies, as well as areas of application, including cytokines [1]. However, the results were also limited to smartphone biosensors for use at the POC in general and not suitable for home usage [1]. The research group identified POC immune biosensors for diseases [1]. For instance, a cellphone-based LFA reader for the detection of malaria, tuberculosis and the human immunodeficiency virus (HIV) suitable for both Android smartphones and iPhone [1,11]. Another example is the combination of a smartphone and microfluidic ELISA chips for the detection of sexually transmitted diseases [1,12,13]. Furthermore, the research group also identified immune biosensors that target cytokines directly [1]. They found the HiSOP platform that uses a smartphone and a 3D-printed microprism for the simultaneous screening of 64 samples by using ELISA to detect IL-6 [1,14]. Another example is the combination of a microplate ELISA assay and a smartphone-based spectrometer to detect levels of IL-6 and a peanut allergen [1,15,16]. The research group also identified immune biosensors that target conditions, e.g., kidney damage, heart failure [1]. For example, a microfluidic ELISA assay was used to detect ovarian cancer and the results were imaged and analyzed via smartphone [1,17].

Hernández-Neuta et al. indicate that the biosensors found are mostly in a proof-of-concept phase and only a few are in the product development phase. The biosensors must still be validated and prove their feasibility in the clinical field [1].

Romeo et al. investigated in their 2016 review [18] the recent advances of multiplexed biosensors and POC diagnostic with smartphones for the measurement of cytokines and other biomarkers [18]. The research group stated that “inexpensive, portable and rapid POC diagnostics for detecting multiple biomarkers […] is feasible nowadays” [18] (p. 5). The need for training of the end user regarding sample preparation is the biggest challenge the research group sees for using the found biosensors [18]. However, once again, the results they found for the detection of cytokine levels are limited for the POC in general and are not suitable for home testing [18]. They reported, for example, on the highly sensitive LSPR biosensor of Chen et al. [19] for the multiplexed detection of six cytokines (IL-2, IL-4, IL-6, IL-10, IFN-γ, TNF-α) within 40 min from 1 µL blood serum [18].

Already in 2015, Chen et al. discussed in their review [20] the performance, potential benefits, challenges, as well as further potential for the development of cytokine biosensors for the rapid determination of the immune status at the POC [20]. Thereby, the research group pointed out the great potential of label-free biosensors [20]. The advantages of label-free biosensors are that the real-time detection of cytokines and the selectivity of the assays can be improved [20]. The “[e]limination of labeling agents […] enables researchers to avoid adverse effects on biomolecular binding events, to achieve quantitative measurements (labeling could be only qualitative due to the inconsistent binding behavior of labels to analytes) and to save cost and time” [20] (p. 6). Furthermore, Chen et al. differentiated the biosensors into mechanical cytokine biosensors, electrochemical cytokine biosensors, optical cytokine biosensors and plasmonic cytokine biosensors [20].

Mechanical cytokine biosensors “detect surface binding events of biological molecules by means of mechanical deflections or dynamic resonance shifts of the transducer system resulting from surface stress or mass changes” [20] (p. 9). The most known representative technique of mechanical cytokine biosensors is the quartz crystal microbalance (QCM) [20]. “The principle of electrochemical biosensing methods is based on an electrical signal change associated with analyte binding onto electroactive sensing elements of a circuit [20]. Correlating the signal change with the quantity of analyte enables the measurement” [20] (p. 11). Representatives of electrochemical cytokine biosensors are, for instance, technologies based on electrochemical impedance spectroscopy (EIS) and nanoelectronic field-effect transistor (FET) [20].

Optical biosensors function in such a way that optical signatures are investigated that arise from the refractive index changes of a solid light-guiding microstructure (e.g., light reflection, transmission or absorbance) upon the surface binding of analytes [20]. Representatives of optical biosensors are silicon photonic microring resonators and photonic crystal resonators [20]. Plasmonic biosensors are about the interaction between electromagnetic radiation on a noble metal in contact with a dielectric medium [20]. More precisely, when the collective motion of the electrons resonates with the incident light of a certain wavelength, electromagnetic modes are created that are confined on the surface [20]. These modes are called “surface plasmons” (SPs) [20]. “SPs emerge under two settings: (1) surface plasmon resonance (SPR), where propagating SPs are excited on a metallic thin film and (2) localized surface plasmon resonance (LSPR), where sub-wavelength-sized metal nanoparticles are excited by an external light source” [20] (p. 18). SPR and LSPR are both representative sensing techniques of plasmonic biosensors [20]. Chen et al. considered biosensors based on FET and LSPR to have the greatest potential for the rapid detection of cytokines at the POC [20]. The research group identified some challenges of the technologies found: low sensitivity, lack of multiplexed detection of cytokines, sensitivity to background noise and slow reaction speed [20].

Further reviews focus on giving an overview of the advances of biosensors at the POC for the detection of cytokines but also other biomarkers (e.g., wearable biosensors [21]) or the relationship between cytokines and a condition (e.g., depression [22,23]).

In summary, we find that some of the reviews either do not focus on the at-home testing or address POC technologies for measuring disease-related biomarkers other than cytokines. In this manuscript, we focus on collecting POC technologies for measuring cytokines at home, possibly with the help of a smartphone, then divide them into technologies that can acquire cytokines inexpensively and those that can acquire them but expensively.

Indeed, cytokines are vital proteins that play a major role in the human body. Therefore, numerous studies have analyzed the correlations between each other and other disease and health conditions. Vculic et al. investigate the correlations among cytokines and age in healthy subjects and among cytokines and age in patients with autoimmune thrombocytopenic [24], whereas the correlations among cytokines, bronchopulmonary dysplasia and modality of ventilation in preterm newborns are presented by Gitto et al. [25]. Hur et al. present how 27 inflammatory cytokines correlate to each other after major burn injury [26].

Furthermore, due to the recent popularity of machine-learning methods, especially in biology and medicine, various studies present machine-learning methods that exploit the measured cytokines to predict the progression of a disease [8,27] or to identify cytokine–protein interactions [9]. In the end, we are not aware of any work that studies the correlations among the cytokines that can be measured easily in a cost-effective way and cytokines that demand highly expensive equipment using machine learning models. The main scope of this study is to propose a cost-effective quantification of cytokines to enable a reliable assessment of the fitness of the immune system.

## 3. Methods I: PRISMA Literature Review

In order to answer the research questions one (RQ_1_) and two (RQ_2_), a systematic literature review is conducted according to PRISMA. PRISMA is the abbreviation for Preferred Reporting Items for Systematic Reviews and Meta-Analyses [28]. PRISMA was designed in 2009 with the aim of making the process of systematic reviews more transparent by stating why and how the review was conducted and what was found [28]. This kind of review method helps readers assess the trustworthiness and applicability of the reviews [28]. Furthermore, it helps readers to replicate the review and update it [28]. PRISMA is a recognized literature research method that has been “widely endorsed and adopted, as evidenced by its co-publication in multiple journals, citation in over 60,000 reports (Scopus, August 2020), endorsement from almost 200 journals and systematic review organizations and adoption in various disciplines” [29] (p. 179). The original PRISMA statement of 2009 was updated in 2020 to keep up with technological developments in conducting systematic literature reviews nowadays [29]. The main component of the PRISMA 2020 statement is a 27-item checklist and was primarily designed for “systematic reviews of studies that evaluate the effects of health interventions, irrespective of the design of the included studies” [29] (p. 180). However, many items of the checklist are applicable for systematic reviews that pursue different purposes [29].

**Figure 1:**
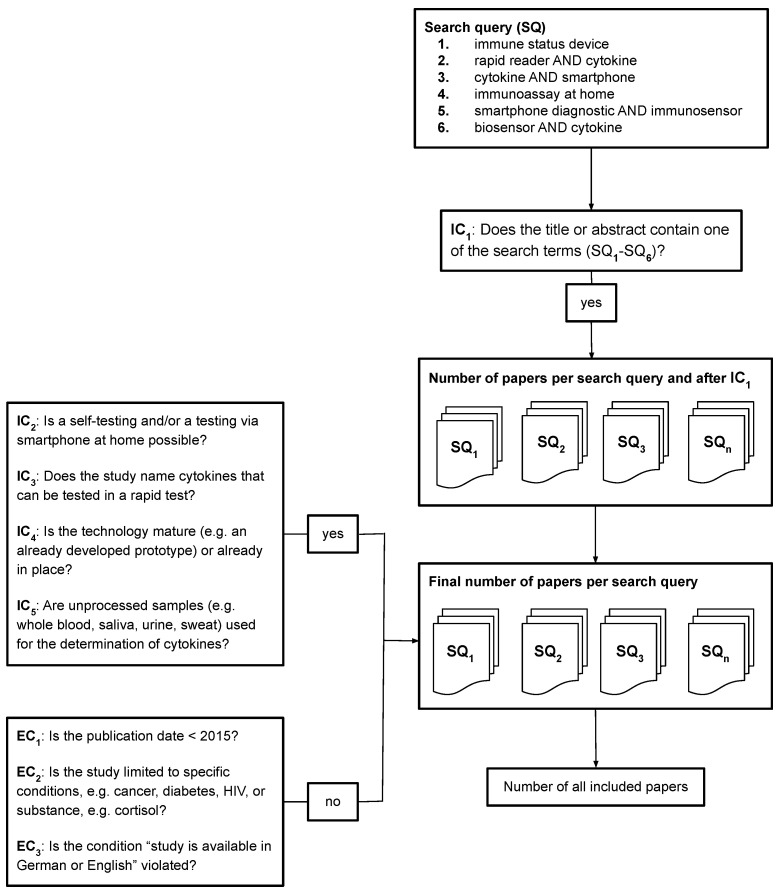
PRISMA literature review process with eligibility criteria. Six search queries (SQs) are used to collect relevant literature. The collected literature should satisfy five inclusion criteria (IC). Any study that violates any of the three exclusion criteria (EC) is filtered out.

For this work, the PRISMA items, eligibility criteria (item 5), information sources (item 6), search strategy (item 7) and study selection (item 16) will be applied. The following is a brief explanation of what the selected PRISMA items contain. Figure 1 illustrates the PRISMA literature research process, the eligibility criteria and the search queries intended for use in this work.

First, the search queries are applied to a total of five databases using the specific search syntax for each database. Inclusion criteria one (IC_1_) has to be already applied in this step, as the syntax for the search in the title and abstract must be adapted accordingly. The identified reports are then assessed for eligibility according to the inclusion and exclusion criteria (IC_2_–IC_5_, EC_1_–EC_3_) presented in Figure 1. Inclusion criteria two (IC_2_) is based on the characteristics for home tests found by Jamaludeen et al. in their review [10]. However, the characteristics are not applied quite as strictly. For instance, measuring multiple cytokines is desirable, but as it is not clear before the PRISMA literature review how many results for home testing even exist, results measuring only one cytokine are also included. The characteristics of small-volume samples in the microlitre range and a rapid assay time will be applied. In case no assay time is specified in the reports, it is also sufficient if the report mentions that the technology is rapid. Furthermore, smartphone-based readers are of most interest in the results, but again it is unclear before the PRISMA literature review how many results this includes. Therefore, reports from technologies that measure cytokines at home using other methods are also included in the results. The fifth characteristic will be applied but extended in inclusion criteria five (IC_5_) for other unprocessed samples such as saliva, sweat or urine. Inclusion criteria three (IC_3_) shows that the review focuses on cytokines. In addition, the technologies identified have to be mature in terms that these are either already in place or tested prototypes already exist (IC_4_). With exclusion criteria one (EC_1_), a year limit for scientific publications not older than 2015 will be applied due to technological developments in recent years (e.g., top-notch cameras in smartphones). Studies that report on technologies that are limited to specific conditions (e.g., diabetes) will be excluded because of exclusion criteria two (EC_2_) as the review intends to find technologies for a general application. Exclusion criteria three (EC_3_) limits the results for German and English languages.

The reports that passed the eligibility criteria are then included in the final results table and the reports that did not pass are dropped.

To answer research question one (RQ_1_), the literature review results are listed. In the overview, all technologies found will be named with the measurable cytokines, the technique behind it, the sample type and, if possible, the sample quantity and sample time. Further, the overview is the basis for answering research question two (RQ_2_), in which the technologies found are to be grouped according to whether the cytokines can be acquired expensively or inexpensively. The results are displayed in two tables: one for cytokines that are expensive to acquire and one table with cytokines that are inexpensive to acquire, each of which also lists the cytokines that are measured with them. The cytokines found, which can be acquired inexpensively through the identified technologies, are then used as independent variables for implementing several regression models for answering the remaining research questions (RQ_3_, RQ_4_).

**Figure 2:**
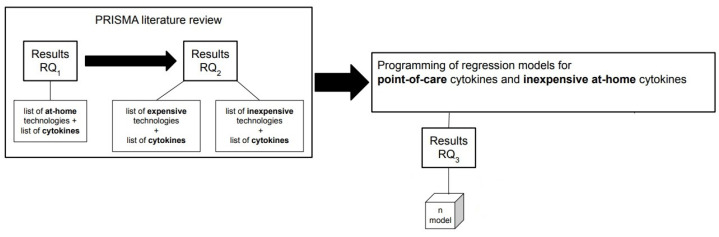
Pictorial overview of the approach to addressing the research questions. After performing the PRISMA literature review, the first research question RQ${}_{1}$ is answered by identifying the POC technologies with the corresponding cytokines that can be measured at home. Then, the at-home technologies with the corresponding cytokines are categorized as expensive and inexpensive cytokines/technologies as an answer to the second research RQ${}_{2}$. Finally, we answer the third research question RQ${}_{3}$ by building machine learning models to predict the expensive cytokines using the inexpensive ones.

Figure 2 illustrates the process from PRISMA literature review to the programming part to answer all research questions (RQ_1_–RQ_4_).

### 3.1. Information Sources

**Figure 3:**
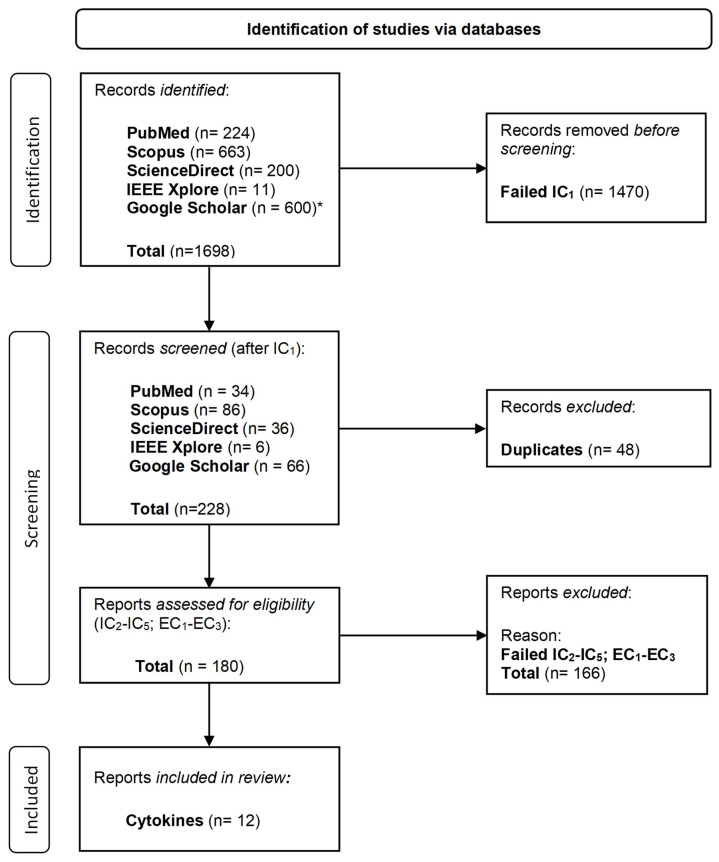
PRISMA flow diagram based on [29] consists of three steps: (1) the identification of relevant literature by the defined search queries (SQs), (2) the screening of all the retrieved literature, namely we keep only papers that satisfy the five inclusion criteria and eliminate papers that satisfy the three exclusion criteria), (3) and the inclusion step ends up with 12 studies found. * Note: A full text search was conducted due to the reasons that a direct abstract search was not possible in Google Scholar and a title search did not return any hits. For each search query there were several thousand hits which could not all be evaluated. Therefore, every search query was limited to the first 10 result pages which equals 100 hits.

The following five databases were searched for papers: PubMed, Scopus, ScienceDirect, IEEE Xplore, and Google Scholar. The IEEE Xplore database had the least hits after IC_1_ was applied (n = 6) (see also Figure 3 for the distribution of hits under the PRISMA item, Study selection in this subsection). This may be due to the fact that this database focuses more on literature in the area of engineering and technology and less on medical literature. As the focus of the literature review is on technical devices in the application field of medicine, it was worth a try to use this database. However, for future literature analyses on this topic IEEE Xplore could possibly be excluded as it did not produce any findings in the end. The other databases produced significantly more results. Scopus produced most hits (n = 86), followed by Google Scholar (n = 66), ScienceDirect (n = 36) and shortly thereafter PubMed (n = 34). The databases were searched between 12 October 2021 and 16 November 2021. Table 1, Table 2, Table 3, Table 4 and Table 5 show the specific access date per search query and the date restrictions that were applied for each database.

### 3.2. Search Strategy

The search queries (SQ_1_–SQ_6_) stated in the introduction were used to conduct the PRISMA literature review.

Table 1, Table 2, Table 3, Table 4 and Table 5 present the website used, the search filters and limits, as well as the access date per search query for each database stated. Language restrictions were not applied as all papers found were in English only. If this had not been the case, a filter for reports in German and English would have been set analogous to exclusion criteria three (EC_3_) of the eligibility criteria.

In the PubMed and IEEE Xplore databases, a search of abstract and title was possible as intended.

In the Scopus and ScienceDirect databases keywords were searched in addition to the abstract and the title, due to the given limitations of search parameters by the databases mentioned.

The search on Google Scholar was slightly different from the other databases as inclusion criteria 1 (IC_1_) could not be fully applied by search parameters. It was possible to conduct a title search but this did not return any hits. An abstract search was not possible. Therefore, a full-text search was conducted but for each search query, there were several thousand hits that could not all be evaluated. Google Scholar is a well-known and well-used database and encompasses all relevant databases, including the other used databases in this research. Therefore, the database was not excluded; instead, every search query was limited to the first 10 result pages which equal 100 hits per search query. Afterward IC_1_ was applied manually. The full-text search seemed to be a good addition to the search in the title and abstract.

### 3.3. Eligibility Criteria

For the identification of suitable papers, inclusion criteria 1 was applied first (IC_1_):

- **IC_1_: Does the title or abstract contain one of the search terms (SQ_1_–SQ_6_)?**

For the realization of IC_1_, the filters and limits stated under PRISMA item Search strategy in this subsection were applied. For the screening of the identified papers, the formulated eligibility criteria were applied.

The papers finally included in the review were then grouped into two categories expensive and inexpensive. Additionally, a third group was found: non-cytokine biomarker, which includes immune biomarkers found that could be tested at home but are not cytokines.

### 3.4. Study Selection

Figure 3 shows a flow diagram of the study selection process. A total of 1698 search hits could be identified with the search in the five databases. The majority of search hits had to be removed after applying IC_1_ (n = 1470). A total of 48 records were duplicates and therefore removed as well while screening the records. In the end, a total of 180 reports were assessed for eligibility. Most of them (n = 166) failed the eligibility criteria (IC_2_–IC_5_, EC_1_–EC_3_). One remark is that none of the retrieved studies reported their results in German. Two more reports were excluded because the tests were limited to animals. Finally, 12 reports remained on technologies that can be used to measure cytokines at home. Not listed among the included reports in the flow diagram are six reports that mention technologies that do not measure cytokines but do measure biomarkers related to the immune system. Therefore, these reports failed IC_3_.

## 4. Results I: Findings of the PRISMA

The findings of the PRISMA literature review make it now possible to answer the research questions one (RQ_1_) and two (RQ_2_):

- RQ_1_: What recent technologies developed for the rapid determination of cytokines are suitable for at-home testing?
- RQ_2_: Which technologies suitable for at-home testing can be used to acquire cytokines inexpensively and which expensively and which cytokines do these technologies measure?

To address RQ_1_, Table 6 and Table 7 show in total 12 technologies developed for the rapid determination of cytokines that can be tested at home. Many of these have only been developed in recent years. For example, most of the technologies are from reports from the years 2020 and 2021. Only four identified technologies were already published in 2018 and 2019. Two possible reasons for this distribution could be the continuous technical and medical progress and the COVID-19 pandemic.

### 4.1. Technologies to Acquire Cytokines Expensively

With their DGTFET platform [30], including a customized Android App and its cloud connection, Hao et al. developed a device for the detection of multiple cytokines. The device consists of an aptameric biosensor chip that is integrated on an electrical device. The biosensor contains one sensing channel on which the target cytokine aptamers are immobilized and another as reference channel to capture changes in environmental conditions. The quantification of the the cytokine is the difference between the signals between the two channels. The cytokines can be detected in several sample types: serum, saliva, urine and sweat [30]. The limits of detection (LODs) achieved by this device for IFN-γ, IL-6 and TNF-α are $476\times 10^{-15}$, $608\times 10^{-15}$ and $611\times 10^{-15}$ M, respectively. The results reported prove the high selectivity of the target cytokines compared to non-target molecules. The wearable device was initially developed for COVID-19 patients, who receive an alert if their condition deteriorates or the patient is at risk of suffering a cytokine storm [30]. At the moment, the device is on the level of a prototype and the acquisition costs for this extra needed device are not yet entirely clear. Therefore, the device was categorized as “expensive”.

The ImmunoFET device [31] from Halima et al. takes samples of saliva to measure the cytokine TNF-α [31]. This device is based on an ion-sensitive field effect transistor (ISFET) which is functionalized with anti-TNF-α antibodies on the gate surface. The detection limit for this device is reported to reach 10–100 fg/mL. The selectivity of the biosensor towards TNF-α is compared to other biomarkers such as N-terminal pro-brain natriuretic peptide (NT-proBNP), interleukin-10 (IL-10) and cortisol. The sample time is not clearly stated. Halima et al. just say that with the device the “non-invasive, rapid and accurate assessment of TNF-α” [31] (p. 1) is possible. The acquisition costs for the extra device to be purchased are not clearly stated as well.

Also for the aptamer-based biosensor from Wang et al., the sample time is not clearly stated [32]. They say their wearable device “can enable the sensitive, rapid and label-free detection of cytokines” [32] (p. 1). With this device, an additional device is needed to measure the cytokine IFN-γ for which the acquisition costs are not mentioned [32]. This device is an aptameric field-effect transistor (GNFET) biosensor device that consists of Graphene–Nafion composite film. The composite structure of the film promotes high sensitive detection of IFN-γ where the limit of detection reported is as low as 740 fM. The high specificity of the biosensors to IFN-γ is proved by analysing the concentration of other inflammatory cytokines TNF-α, interleukin-002 (IL-002) and inter-leuk-6 (IL-6). A prototype is available of the device.

Wang et al. developed another GFET biosensor that was manufactured on a bio-compatible and ultra-thin, polymer-supporting substrate [33] in 2020 to measure IFN-γ and TNF-α of human biofluids like tears, sweat or saliva in just seven minutes. The limits of detection achieved for TNF-α and IFN-γ are down to 2.75 and 2.89 pM, respectively. The biosensor achieves high specificity when the detection of the target cytokines is compared to the detection of other control proteins, EGF and GH. According to the report, the biosensor has so far only been tested with artificial tears and acquisition costs or extra equipment needed are not exactly described. However, a prototype is available [33].

The QCM biosensor [34] of Pohanka is fabricated on piezoelectric principles and uses gold nano-particles. This immunoassay needs a longer sample time (90 Mins) compared to the other expensive technologies to measure IFN-γ and TNF-α from samples of whole blood, blood plasma or blood serum [34]. The limit of detection is down to 5.7 pg/mL. Pohanka stated that the device “was constructed as a tool suitable for small laboratories or home care conditions” [34] (p. 4) and its “major advantage […] is overall simplicity, low cost per one assay, suitability for mass production of the biosensor devices, low volume of sample and no special requirements […]” [34] (p. 4). The distinction between expensive and inexpensive cytokines was difficult here, as the device itself seems to be inexpensive, but the report does not explain how the results are read and whether a separate device is needed for this. Since it cannot be said with certainty here that no further device is needed, Pohanka’s biosensor was assigned to the technologies where cytokines can only be acquired expensively.

### 4.2. Technologies to Acquire Cytokines Inexpensively

The paper biosensor [35] of Adrover-Jaume et al. is interfaced with a smartphone and was initially developed for detecting severe cases of COVID-19 but is also applicable to other areas. The paper sensor generates “intense colorimetric signals when the sample contains ultra low concentrations” [35] (p. 1) of the cytokine IL-6. “This is achieved by combining a paper-based signal amplification mechanism with polymer-filled reservoirs for dispensing antibody-decorated nanoparticles and a bespoken app for color quantification” [35] (p. 1). The limit of detection achieved for IL-6 is as low as 1.3 pg/mL. The device has so far only been tested with IL-6, but according to Adrover-Jaume et al. the device “could be easily adapted to detect other cytokines such as TNF-α and IL-8” [35] (p. 1). The device uses samples of whole blood or respiratory samples and the readout of the result via smartphone is available in under ten minutes [35]. The report does not state the amount of blood used but the pictures in the report indicate that it might be just a few drops of blood.

Punj et al. developed a self-assembled monolayer (SAM) multiplex electrochemical biosensor [36] that measures IL-6 on samples of tears and blood plasma in 20 min. The SAM technique improves the specificity and the sensitivity of the biosensor. The limit of detection of the IL-6 is estimated to be 22.1 pg/mL. So far, the device has only been tested on artificial tears [36]. Punj et al. promote their device with the words “[the] biosensor simplifies the detection procedure, shortens the detection time, implements only simple electronic interfaces, requires a small sample volume and is cost-effective” [36] (p. 6). A prototype of the device has already been developed.

Alba-Patiño et al. developed in 2020 a plasmonic-based mobile biosensor [37] made of filter paper for the detection of IL-6 in whole blood. The cytokine is measured with the help of a custom app that “makes use of a software-based AR guidance system” [37] (p. 1258) “in order to stabilize angle and distance parameters” [37] (p. 1258) and an unmodified smartphone. For this, it is only necessary to hover over the assay with the smartphone to quantify colorimetric signals [37]. This immunoassay could detect IL-6 with a limit of detection of 0.1 pg/mL.

Another electrochemical biosensor that is based on an impedance spectroscopy (EIS) system [38] was developed by Cruz et al. for measuring TNF-α in samples of human blood serum, cerebrospinal fluid and tears. Cruz et al. state that the device is also applicable “in the context of personalized medicine” [38] (p. 2676) and could be further developed for other immune relevant biomarkers. The benefits of the device are “high sensitivity and reproducibility, its ease of use, low processing and signal acquisition times” [38] (p. 2680). The estimated limit of detection (LOD) is as low as 0.085 pg/mL. This immunoassay demonstrates the high selectivity to TNF-α in a solution that contains IL-4 and IFN-γ. Compared to the other technologies that can measure cytokines inexpensively, with 90 min the device needs more time to present the result [38]. There is already a prototype available which has similarities to a glucose meter that will be connected to a computer.

A graphene-based portable nanosensing system [39] was developed by Hao et al. in 2019 to detect levels of IL-6 in saliva in just about 400 s with a limit of detection as low as 12 pM. The biosensor can be used in combination with a smartphone: “The signal can be wirelessly transmitted to a smartphone or cloud sever through the Wi-Fi connection for visualizing the trend of the cytokine concentration change” [39] (p. 1). A customized app was developed specifically for this purpose [39]. A prototype is available of the nanosensing system and was tested with an Android smartphone [39].

Barbosa et al. developed an MCF device based on enhancing enzymatic amplification [40] to detect the level of IL-1β in whole blood with the usage of “fluoropolymer microfluidic MCF strips” [40] (p. 319) and with a detection limit of 6 pg/mL. Barbosa et al. use cost effective optoelectronic readout systems such as smartphones and a flatbed scanner [40]. The readout time was not clearly stated, the researchers just stated several times that the device is for the rapid determination of the cytokine [40]. The device is available at a prototype level [40].

A hybrid magneto-electrochemical sensor [41] was developed by Min et al. for the detection of levels of IL-3 in whole blood, blood plasma and serum under 60 min. For samples of whole blood, no pre-processing steps are needed [41]. The achieved detection sensitivity and specificity are 91.3% and 82.4%, respectively. The sensor is used in combination with a smartphone that functions “as a touch-screen interface” [41] (p. 3). Additionally, Min et al. developed a customized iOS app “for system control and data storage” [41] (pp. 3–4). “The resulting […] device was a standalone handheld unit, measuring electrical currents and displaying IL-3 concentrations” [41] (p. 4). The cost of the device is 50 USD [41]. In the future, the researchers intend to work to extend the device to measure multiple cytokines, such as IL-3, TNF-α, IL-1β and IL-6 [41].

In order to answer RQ_2_, Table 6 shows the technologies that can be used to acquire cytokines expensively and Table 7 presents the technologies with which cytokines can be acquired inexpensively. Furthermore, in the tables, the cytokines that can be measured are shown for each technology. Hence, the cytokines that can be measured at home are: IL-1β, IL-3, IL-6, IL-8, TNF-α and IFN-γ.

Interleukin 1β (IL-1β) is a cytokine that is “critical for B-cell maturation, stimulation of T-cells, activation of natural killer (NK) cells and activation of macrophages and microglia, leading to inflammatory responses” [42] (p. 922) and plays a critical role in the severity of inflammation [42]. Interleukin 3 (IL-3) is “a cytokine that activates proliferation of hematopoietic stem cells and progenitors, has recently been identified as a key regulator during sepsis pathogenesis. IL-3 operates upstream of key cytokines including TNF-α, IL-1β and IL-6; high IL-3 level can trigger a detrimental cytokine storm” [41] (p. 5). Interleukin 6 (IL-6) has two important roles. On the one hand, IL-6 is a pro-inflammatory cytokine and on the other hand it is an anti-inflammatory cyokine [43]. Furthermore, it is “an important mediator of fever” [43] (p. 5). Interleukin 8 (IL-8) is a pro-inflammatory cytokine and is responsible for chemoattraction and neutrophils activation [44]. Tumor necrosis factor alpha (TNF-α) is a major inflammatory mediator and is responsible for many of the physical symptoms that occur during the inflammatory process [38]. TNF-α is seen as the gold-standard biomarker for inflammation [31]. Interferon gamma (IFN-γ) “can be used for revealing of infectious diseases and especially for distinguishing of viral and some types of bacterial infections” [34] (p. 1).

Table 8 summarises which of the mentioned cytokines are inexpensive and which expensive to acquire.

### 4.3. Technologies to Acquire Non-Cytokine Biomarkers

C-Reactive Protein (CRP), Prostate Specific Antigen (PSA) as well as both antibodies Immunoglobulin G (IgG) and ImmunoglobulinM (IgM) have also been observed during the literature review and are listed in Table 9. These biomarker are also related to the immune system but are no cytokines. Gondoh-Noda et al. state that “C-Reactive Protein (CRP) is highly sensitive to diagnosing infections and its elevation can help diagnose acute infection in older patients” [45] (p. 1). Normally, the immune status is assessed by a whole blood count with cell differential but in some cases also the determination of the level of CRP is carried out in addition to this [46].

Bouraoui et al. were able to identify an association between elevated proinflammatory cytokines (TNF-α, IL-6, IL-1) and elevated serum PSA levels with regard to progression of prostate cancer [47]. The MCFphone in Table 9 measures PSA and “is composed by a smartphone integrated with a magnifying lens, a simple light source and a miniaturized immunoassay platform, the Microcapillary Film (MCF)” [48] (p. 1).

Choi et al. developed the Eigen Diagnosis Platform (EDP) as “a portable quantitative immunoassay platform” [49] and demonstrated the performance of the platform for IgG and IgM. The IgG antibodies play an important role in fighting both bacterial and virus-related infections and IgM is usually the first antibody response to an infection [49]. The monitoring of both antibody levels can help in the diagnosis and treatment of health conditions [49].

**Table 9:** Findings of the PRISMA literature review for six non-cytokine biomarker.

Year	Device(s)	Biomarker	Technique	Sample	Sample Time
2020	GLEIA device [45]	CRP	GLEIA	1.4 µL of whole blood	<10 min
2017	PhoneQuant [50]	CRP	LFIA	whole blood	−
2017	iQuant™Analyser [51]	CRP	LFIA	whole blood, serum	1 min
2016	EE-*µ*PAD [52]	CRP	paper-based, electronic and opto-electronic sensors	single drop of whole blood, sweat, saliva	few min
2016	Eigen Diagnosis Platform and App, smartphone [49]	IgG, IgM	GMR	single drop of whole blood	<15 min
2015	MCFphone	PSA	MCF	whole blood	13 to 22 min

## 5. Dataset

**Figure 4:**
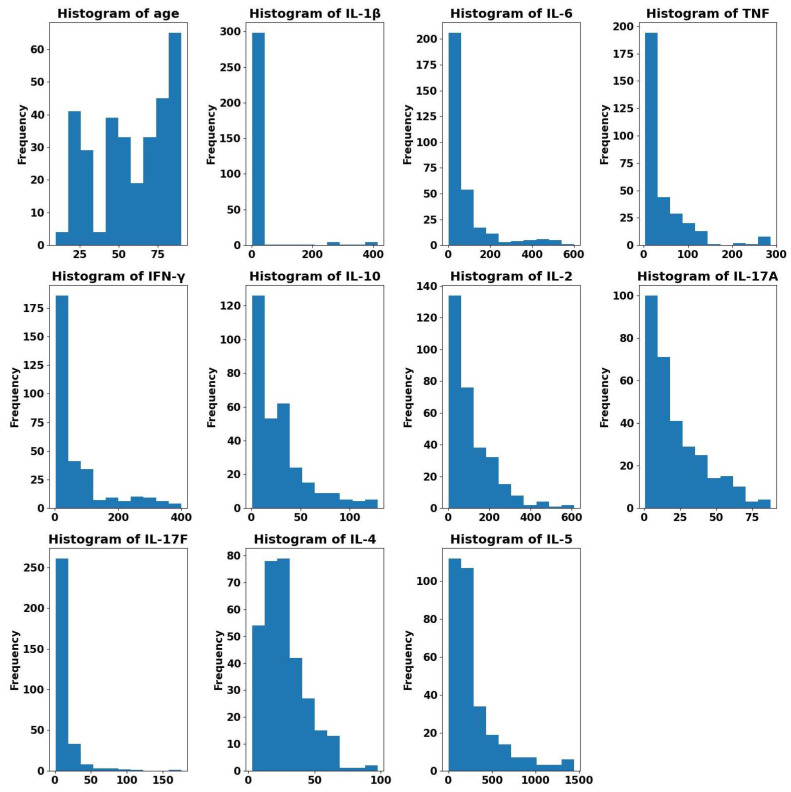
Sample distributions (after the removal of outliers) for all the ten expensive and inexpensive cytokines, IL-1β, IL-6, TNF, IFN-γ, IL-10, IL-2, IL-17A, IL-17F, IL-4 and IL-5 in addition to the age of 351 healthy subjects in the public dataset 10k Immunomes. Most of the cytokines do not follow a normal distribution.

We use a public dataset of healthy human subjects collected for analyzing the immunological mechanisms in the immune system [53] to answer the research question RQ${}_{3}$. The dataset comprises measurements of 42 immunological proteins of 10,000 healthy subjects. The measurements were performed by using multiplex ELISA. After removing the missing values and outliers, we end up with 351 healthy subjects where we only focus on ten cytokines, IL-1β, IL-6, TNF, IFN-γ, IL-10, IL-2, IL-17A, IL-17F, IL-4 and IL-5. According to the results of the PRISMA in Section 3, {IL-1β, IL-6, IL-3, IL-8 and TNF} are categorized as inexpensive cytokines and the rest as expensive since they only can be measured with additional complex equipment. We do not use IL-3 and IL-8 in the regression analysis because IL-3 is missing for more than 98% of the subjects and IL-8 is not measured in this dataset. Moreover, we add age to the inexpensive cytokines since this information is available in the dataset and easy to collect. Figure 4 shows the value distribution of each of the ten cytokines in addition to the age after the removal of outliers.

## 6. Method II: Regression Analysis

To answer the third research question, the cytokines summarized in Table 8 are needed because these cytokines form the basis for the implementation of several regression models. In order to answer research question three (RQ${}_{3}$), the cytokines that are inexpensive to acquire are used as independent variables (the features) and the cytokines that are expensive to acquire are used as dependent variable (the target). Three different types of regression algorithm will be selected and the results of the regression models will be later compared with each other. In this process, three regression models per selected regression algorithm are developed.

### 6.1. Regression Models

The development of the regression models is implemented with the programming language Python.

Three regression algorithms with their different advantages and disadvantages were chosen for the training of regression models based on the public dataset 10k Immunomes: (multiple) linear regression, decision tree and random forest.

#### 6.1.1. (Multiple) Linear Regression

Multiple linear regression concentrates on the linear relationship between the dependent target variable and more than one independent input variable [54]. Therefore, one assumption for using multiple linear regression is linearity which means if the relationship between any of the input variables and the target variable is non-linear the model performance is not as accurate as it could be [55]. Another assumption that has to be kept in mind is the normal distribution regarding residuals [55].

The (multiple) linear regression algorithm is easy to understand and to implement but it also has some downsides [56]. Linear regression models tend to overfit and to simplify complex relationships between variables too much [56]. Furthermore, as the name already implies, it cannot work on non-linear relationships [56]. Outliers can have a large effect on the performance of a linear regression model [55].

#### 6.1.2. Decision Tree

In contrast, the second selected regression algorithm is the decision tree algorithm which is an example of a non-linear method.

The biggest advantage of the decision tree algorithm is that it can handle next to non-linear data and also linear data and compared to other algorithms a decision tree is quick to train [57]. Furthermore, it is easy to interpret and to visualise [58]. The biggest disadvantage is its tendency to overfit [56].

#### 6.1.3. Random Forest

A possibility to avoid overfitting is to use the random forest model. Here, various decision trees are generated to combine the predictions of them [59].

The random forest algorithm generates randomly several training datasets from the original dataset by using the bootstrapping method [60]. The method is related to bagging (bootstrapping aggregation) which is used to aggregate data for making predictions on the basis of multiple build models on a subset of data [60]. In the end all predictions will be the mean value of the predictions of all decision trees [60].

All three models presented are very different in their approach. They have different strengths and weaknesses, with some weaknesses of one algorithm being mitigated by the approach of another of the selected algorithms (e.g., the tendency of a decision tree to overfit is prevented by the random forest). It is to be expected that the selected regression algorithms will perform differently and produce different “good” models. With this in mind, a comparison of these models seems very interesting.

### 6.2. Data Preprocessing Steps

It is important to conduct pre-processing of the data before building the regression models. In [61], Manasa et al. describe the general steps in pre-processing of the data: the handling of missing values, the splitting of the dataset into a training set and a test set, the scaling of variables, feature selection and the handling of outliers. In addition, a Shapiro–Wilk Test for normal distribution is to be carried out and a heatmap for the identification of multi-collinearity is created to account for the assumptions of (multiple) linear regression. We decide not to impute the missing values and simply remove them because the imputation of missing values may introduce bias into the data. For evaluation, we apply the hold-out validation with random sampling with no replacement over 40 runs. The dataset is divided into training (80% of the data) and testing sets (20% of the data). The measurements are also standardized, the features have different scales. This can affect the predictor and features with bigger scales may get more weight.

#### 6.2.1. Feature Selection

Another essential step in preparing the data for a later model is feature selection. Since the aim of this work is to find out whether the identified features from the results of research questions one (RQ${}_{1}$) and two (RQ${}_{2}$) can predict the expensive cytokines, none of the features are eliminated. However, to identify which inexpensive cytokines contribute to the prediction of the expensive cytokines, we investigate the performance of various regression models built on different subsets of the inexpensive cytokines. Since we have four inexpensive cytokines, IL-6, IL-1β, TNF and age, we create 15 different sets of all possible combinations as follows: {IL-6}, {IL-1β}, {TNF}, {age}, {IL-6, IL-1β}, {IL-6, TNF}, {IL-6, age}, {IL-1β, TNF}, {IL-1β, age}, {TNF, age}, {IL-6, IL-1β, TNF}, {IL-6, IL-1β, age}, {IL-1β, TNF, age}, {IL-6, TNF, age}, {IL-6, IL-1β, TNF, age}. Afterward, for each of the dependent variables (expensive cytokines), we get the top 10 regression models that perform the best. Then, we check which of the inexpensive cytokines are used as independent variables by these top 10 models. We consider the most important inexpensive cytokines to be those that appear frequently in the lists of the independent variables of the top 10 models.

#### 6.2.2. Shapiro–Wilk Test

Before feature scaling and removing outliers, a Shapiro–Wilk Test is conducted to check if the data come from a normal distribution which would help to meet the assumption of normally distributed data for the linear regression.

For all the cytokines, the *p*-value is less than 0.05, which means it is very likely to reject the null hypothesis given the available evidence and that the sample data does not stem from a normal distribution. The absence of a normal distribution can affect the predictor of Linear Regression. Figure 4 proves that all the features in the 10k Immunomes dataset do not follow a normal distribution.

#### 6.2.3. Multi-Collinearity

As the Linear Regression has the assumption that there is no multi-collinearity between the independent variables, a heatmap using the Pearson correlation is plotted to investigate this.

**Figure 5:**
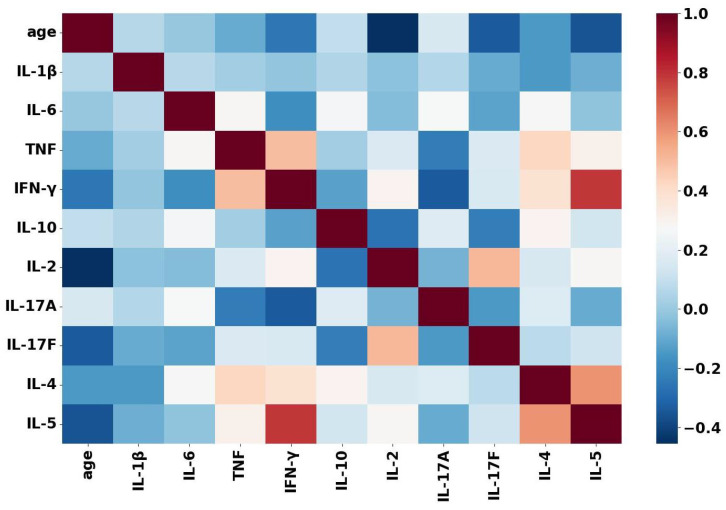
A heatmap of the Pearson correlation coefficients among the inexpensive and the expensive cytokines, in addition to ‘age’ as an independent variable in the linear and nonLinear regression models. The heatmap illustrates weak correlations among the four independent variables, IL-1β, IL-6, TNF and age; IL-5 is strongly positively correlated with both IFN-γ and IL-4 and TNF is strongly negatively correlated with IFN-γ.

According to the Pearson correlation coefficients shown in Figure 5, there are very low correlations among the variables. The four independent variables (the three inexpensive cytokines (IL-1β, IL-6, TNF) in addition to the ‘age’) are weakly correlated because the correlation coefficients range from −0.2 to 0.2. Thus, the multi-collinearity poses no threat to the validity of the linear regression models. Furthermore, the correlation heatmap reveals some strong correlations between the expensive cytokines. We find that IL-5 is strongly correlated with the two expensive cytokines IFN-γ and IL-4. Moreover, TNF and IFN-γ are strongly negatively correlated.

#### 6.2.4. Outliers

**Figure 6:**
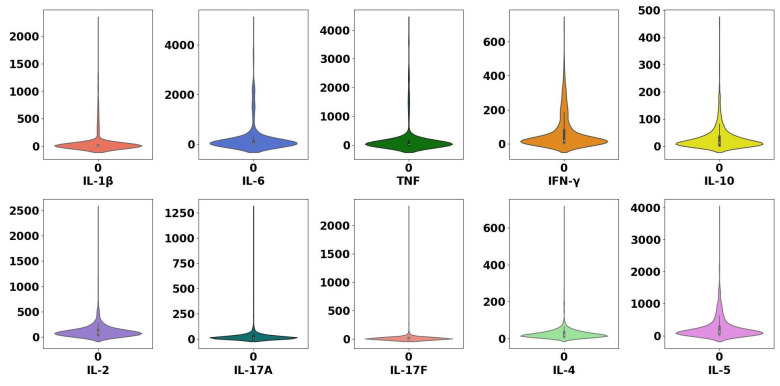
Violin plot of all cytokines in the public dataset 10k Immunomes before the removal of outliers. The graph indicates the existence of outliers for all the cytokines due to the extremely wide range of values.

**Figure 7:**
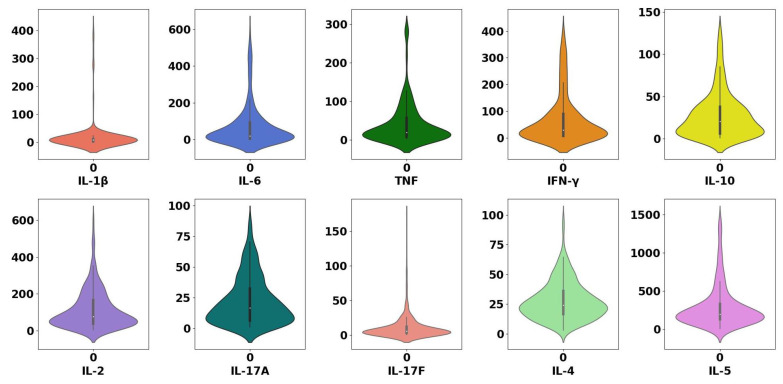
Violin plot of all cytokines in the public dataset 10k Immunomes after the removal of outliers. The removal of outliers shrinks the range of values.

There are several reasons for possible outliers, e.g., mistakes during the data collection, recording or transcription [62]. With the help of violin plots, possible outliers can be detected. The dataset used has outliers that may affect the model building as illustrated in Figure 6. All the subjects in this dataset are healthy subjects. Therefore, we consider highly elevated cytokine values as outliers. For example, while most of the values of IL-6 are concentrated below 1000 pg/mL, some of them are higher than 4000 pg/mL. Thus, we consider all values that are higher than 4000 pg/mL for IL-6 as outliers. Similarly, for the cytokines IL-1β, IFN-γ, TNF, IL-10, IL-2, IL-17A, IL-17F, IL-4 and IL-5, we consider the values that are higher than 500, 500, 300, 200, 700, 100, 200, 100 and 1500 pg/mL, respectively, as outliers and hence we remove them. Figure 7 shows the value ranges for all the cytokines after the removal of the outliers.

Another part of the implementation is the hyperparameter tuning to improve the models’ performance and accuracy. Especially for random forest models and decision tree models, different hyperparameters can be tuned (e.g., the minimum number of samples required to split an internal node, the maximum depth of a tree, the minimum number of samples required to build a leaf node, the number of trees in a forest) [63]. The hyperparameters that improve the performance (e.g., the highest possible value for the coefficient of determination) of the regression models are used in the final implementation. The search for hyperparameters was carried out manually. For the random forest regressor, we test several numbers of learned trees (20, 35, 30, 40 and 50). The depth of the learned trees in the random forest and the decision tree regressors is varied between the number of the independent variables and the number of the independent variables + 3. Furthermore, to compare the performance of the several regression models, we must choose the evaluation measure carefully. There are several evaluation metrics that are used in regression models, such as mean absolute error (MAE), root mean of squared errors (RMSE), the sum of squared errors (SSE) and the r-squared score of determination ($R^{2}$). We choose to use the score of determination because it evaluates how the variance of the dependent variable can be explained by the model. The value of this measure is between 0 and 1 and it is independent of the value scale of the variable, unlike all the remaining evaluation measures that are not scale-free.

## 7. Results and Discussion II: Findings of the Regression Analysis

The aim of the implementation of different regression models was to answer research question three (RQ${}_{3}$). Therefore, the findings of the implementation are presented below as well as the discussion of the findings. For the random forest regression model, we find that the models with a maximum depth of the tree that equals the number of the independent variables +3 and 30 randomly generated trees give the best performance. Moreover, for the decision tree regression model, the best results are produced by trees with a maximum depth equals to the depth chosen for the random forest regression model.

As the results show in Table 10, the random forest regression model dominates in terms of exhibiting the best performance across all the expensive cytokines. We can say that among all the expensive cytokines, IFN-γ, IL-5 and IL-4 can be predicted to be the best with random forest models that achieve $R^{2}=$ 0.743, 0.69, 0.517, respectively. The best decision tree regression models that predict IFN-γ, IL-4 and IL-5 score $R^{2}$ = 0.76, 0.561, 0.336, respectively, which is higher than the best predictions achieved by the Linear Regression models ($R^{2}$ = 0.373, 0.183, 0.195, respectively). This emphasizes the non-linear dependencies between these expensive cytokines and the inexpensive ones.

The best predictor for IL-10 achieves the lowest $R^{2}$ (=0.126) among all the expensive cytokines which implies that it is difficult with the available four inexpensive cytokines to generate good predictions of IL-10.

Furthermore, not all the four inexpensive cytokines are needed to achieve the best predictions. The best predictors of IFN-γ and IL-4 use only TNF, IL-6 and IL-1β as independent variables whereas age, TNF and IL-6 are the independent variables used by the best predictor of IL-5. Similarly, the best predictor of IL-2 uses the same independent variables used be the best predictor of IL-5. The best regression models that predict the expensive cytokines, IL-10, IL-17A and IL17F, need all the four inexpensive cytokines. As a result, we note that TNF appears among the inexpensive cytokines that predict all the expensive cytokines. This indicates the high importance of the cytokine TNF in the immune system. These findings are supported with the estimated feature importance on the top 10 prediction models for each of the expensive cytokines, as shown in Table 11. We find that TNF appears as an independent variable in all the top 10 models for predicting three cytokines, IFN-γ, IL-4 and IL-5. IL-6 appears in all of the top 10 prediction models of the cytokine IL-10 and in 9 out of 10 of the top 10 models for predicting IL-17A. Age is also significantly important as it is among the independent variables for all the top 10 prediction models for IL-2 and in 8 out of 10 of the top 10 prediction models for IL-17F.

Moreover, we observe that selecting the independent variables plays a major role in producing reliable predictions. For example, the random forest model that uses the age, IL-6 and IL-1β for predicting IFN-γ scores an $R^{2}$ equals to 0.069, which is improved to 0.743 by using the same random forest model that replaces age with TNF. It is evident that the strong correlation between IFN-γ and TNF, shown in Figure 5, leads to more precise predictions. In most cases, the linear and the decision regression models perform poorly. We do not observe any consistent pattern in their performance. This could be due to the complex dependencies among the cytokines or because the data are not normally distributed, as we discussed earlier in Section 6.2.

## 8. Conclusions

In this review, we identified in total 12 technologies developed for the rapid determination of cytokines that can be tested at home. Most of the technologies found are from recent years (2018 to 2021) and are still in the prototype phase. Additionally, further technologies could be identified that measure no cytokines but other immune-related biomarkers. The identified technologies were divided into inexpensive and expensive technologies depending on whether the cytokines are easy and inexpensive to acquire with these technologies or not. Inexpensive-to-acquire cytokines suitable for home testing could be identified: Interleukin 1β (IL-1β), Interleukin 3 (IL-3), Interleukin 6 (IL-6), Interleukin 8 (IL-8) and Tumor necrosis factor alpha (TNF-α). The only expensive-to-acquire cytokine suitable for home testing identified is Interferon gamma (IFN-γ).

The PRISMA literature review results were the basis for answering research question three (RQ${}_{3}$) as the identified inexpensive-to-acquire cytokines are the input variables and the expensive-to-acquire cytokine IFN-γ in addition to six cytokines from the public dataset 10k Immunomes are the target variable for the implementation of regression models. Three different regression algorithms were implemented for a group of 351 healthy subjects: (multiple) linear regression, decision tree and random forest. With the selected algorithms, the implemented approach and the available dataset, the following results could be achieved: the best performing model is the random forest model for the expensive cytokine IFN-γ with $R^{2}=0.743$, followed by IL-5 then IL-4 with $R^{2}=0.69$, 0.517 respectively. TNF had a significant role in the immune system as it appears among the independent variables used for predicting all the seven expensive cytokines. The regression analysis results also show that it is difficult to predict the expensive cytokine IL-10 relying solely on the identified inexpensive cytokines.

This study focuses on modeling the dependencies between the expensive and the inexpensive cytokines in healthy subjects. However, it is also necessary to analyse these dependencies in subjects with some health conditions because we expect these dependencies to differ when the studied subjects have a disease.

Quantifying the cytokines that play vital roles in the immune system allows for a reliable assessment of the immune system’s fitness and the body’s health. Therefore, developing an efficient method that does not require many resources is necessary. We believe that exploiting the inexpensive cytokines to quantify the expensive ones moves us one step closer to assessing the fitness of the immune system without the need for complex equipment and professional staff.

## Figures and Tables

**Table 1:** Search limits and filters for the database PubMed. Six search queries (SQ${}_{1}$–SQ${}_{6}$) are used in addition to a restriction of the publication year from 2015 to 2021.

	PubMed
**Website**	www.pubmed.gov (accessed on 15 November 2021)
**SQ${}_{1}$**	immune status device [Title/Abstract]
**SQ${}_{2}$**	rapid reader [Title/Abstract] AND cytokine [Title/Abstract]
**SQ${}_{3}$**	cytokine [Title/Abstract] AND smartphone [Title/Abstract]
**SQ${}_{4}$**	immunoassay at home [Title/Abstract]
**SQ${}_{5}$**	smartphone diagnostic [Title/Abstract] AND immunosensor [Title/Abstract]
**SQ${}_{6}$**	biosensor [Title/Abstract] AND cytokine [Title/Abstract]
**Further restrictions**	2015–2021
**Access date**	15 November 2021 (SQ${}_{1}$–SQ${}_{6}$)

**Table 2:** Search limits and filters for the database IEEE Xplore. Six search queries (SQ${}_{1}$–SQ${}_{6}$) are used in addition to a restriction of the publication year from 2015 to 2021.

	IEEE Xplore
**Website**	https://ieeexplore.ieee.org (accessed on 16 November 2021)
**SQ${}_{1}$**	(“Publication Title”: immune status device) OR (“Abstract”: immune status device) Filters Applied: 2015–2021
**SQ${}_{2}$**	(“Publication Title”: rapid reader) AND (“Publication Title”: cytokine) OR (“Abstract”: rapid reader) AND (“Abstract”: cytokine) Filters Applied: 2015–2021
**SQ${}_{3}$**	(“Publication Title”: cytokine) AND (“Publication Title”: smartphone) OR (“Abstract”: cytokine) AND (“Abstract”: smartphone) Filters Applied: 2015–2021
**SQ${}_{4}$**	(“Publication Title”: immunoassay at home) OR (“Abstract”: immunoassay at home) Filters Applied: 2015–2021
**SQ${}_{5}$**	(“Publication Title”: smartphone diagnostic) AND (“Publication Title”: immunosensor) OR (“Abstract”: smartphone diagnostic) AND (“Abstract”: immunosensor) Filters Applied: 2015–2021
**SQ${}_{6}$**	(“Publication Title”: biosensor) AND (“Publication Title”: cytokine) OR (“Abstract”: biosensor) AND (“Abstract”: cytokine) Filters Applied: 2015–2021
**Further restrictions**	Specify Year Range: From 2015 to 2021
**Access date**	16 November 2021 (SQ${}_{1}$–SQ${}_{6}$)

**Table 3:** Search limits and filters for the database Scopus. Six search queries (SQ${}_{1}$–SQ${}_{6}$) are used in addition to a restriction of the publication year from 2015 to 2021.

	Scopus
**Website**	www.scopus.com (accessed on 12–20 October 2021)
**SQ${}_{1}$**	TITLE-ABS-KEY (immune AND status AND device) AND PUBYEAR > 2014
**SQ${}_{2}$**	TITLE-ABS-KEY (rapid AND reader AND cytokine) AND PUBYEAR > 2014
**SQ${}_{3}$**	TITLE-ABS-KEY (cytokine AND smartphone) AND PUBYEAR > 2014
**SQ${}_{4}$**	TITLE-ABS-KEY (immunoassay AND at AND home) AND PUBYEAR > 2014
**SQ${}_{5}$**	TITLE-ABS-KEY (smartphone AND diagnostic AND immunosensor AND PUBYEAR > 2014)
**SQ${}_{6}$**	TITLE-ABS-KEY (biosensor AND cytokine) AND PUBYEAR > 2014
**Further restrictions**	2015–2021
**Access date**	12 October 2021 (SQ${}_{1}$), 19 October 2021 (SQ${}_{2}$, SQ${}_{3}$), 20 October 2021 (SQ${}_{4}$–SQ${}_{6}$)

**Table 4:** Search limits and filters for the database ScienceDirect. Six search queries (SQ${}_{1}$–SQ${}_{6}$) are used in addition to a restriction of the publication year from 2015 to 2021.

	ScienceDirect
**Website**	www.sciencedirect.com (accessed on 16 November 2021)
**SQ${}_{1}$**	Title, abstract, keywords: immune status device
**SQ${}_{2}$**	Title, abstract, keywords: rapid readers AND cytokine
**SQ${}_{3}$**	Title, abstract, keywords: cytokine AND smartphone
**SQ${}_{4}$**	Title, abstract, keywords: immunoassay at home
**SQ${}_{5}$**	Title, abstract, keywords: smartphone diagnostic AND immunosensor
**SQ${}_{6}$**	Title, abstract, keywords: biosensor AND cytokine
**Further restrictions**	2015–2021
**Access date**	16 November 2021 (SQ${}_{1}$–SQ${}_{6}$)

**Table 5:** Search limits and filters for the database Google Scholar. Six search queries (SQ${}_{1}$–SQ${}_{6}$) are used in addition to a restriction of the publication year from 2015 to 2021.

	Google Scholar
**Website**	https://scholar.google.com (accessed on 16 November 2021)
**SQ${}_{1}$**	immune status device
**SQ${}_{2}$**	rapid readers AND cytokine
**SQ${}_{3}$**	cytokine AND smartphone
**SQ${}_{4}$**	immunoassay at home
**SQ${}_{5}$**	smartphone diagnostic AND immunosensor
**SQ${}_{6}$**	biosensor AND cytokine
**Further**	2015–2021, first ten result pages due to several thousand hits
**restrictions**	for every search query
**Access date**	16 November 2021 (SQ${}_{1}$–SQ${}_{6}$)

**Table 6:** Findings of the PRISMA literature review for five expensive-to-acquire cytokines.

Year	Device(s)	Cytokine(s)	Technique	Sample	Sample Time
2021	DGTFET biosensing device + customized Android App + cloud connection [30]	IFN-γ, TNF-α, IL-6	DGTFET	serum, saliva, urine, sweat	7 min
2021	ImmoFET [31]	TNF-α	ISFET, EIS	saliva	−
2021	aptamer-based biosensor [32]	IFN-γ	GFET	undiluted human biofluids, e.g., sweat	−
2020	GFET biosensor [33]	IFN-γ, TNF-α	GFET	40 µL of human biofluids, e.g., tears, sweat, saliva	7 min
2020	QCM biosensor [34]	IFN-γ, TNF-α	QCM, gravimetric analysis	50 µL of blood, blood plasma, serum	90 min

**Table 7:** Findings of the PRISMA literature review for seven inexpensive-to-acquire cytokines.

Year	Device(s)	Cytokine(s)	Technique	Sample	Sample Time
2021	paper immunosensor interfaced with a smartphone [35]	IL-6, IL-8, TNF-α	paper-based, app for color quantification	whole blood and respiratory samples	<10 min
2020	electrochemical biosensor [36]	IL-6	SAM of alkanethiols on gold	tears, blood plasma	20 min
2020	plasmonic-based mobile biosensor [37]	IL-6	LSPR	2.5 µL of whole blood	17 min
2019	electrochemical biosensor [38]	TNF-α	EIS	blood serum, cerebrospinal fluid, tears	90 min
2019	GFET nanosensing system [39]	IL-6	GFET	saliva	400 s
2018	MCF device [40]	IL-1β	MCF	whole blood	−
2018	magneto-electro-chemical sensor [41]	IL-3	magneto-electro-chemical	100 µL of whole blood, plasma, serum	<60 min

**Table 8:** The identified inexpensive- and expensive-to-acquire cytokines.

Inexpensive	Expensive
IL-1β	IFN-γ
IL-3	
IL-6	
IL-8	
TNF-α	

**Table 10:** Results of the performance indicator ($R^{2}$) of the three regression models (Linear regression, random forest regression (RF), decision tree regression (DT)) for all the cytokines in the public dataset 10k Immunomes across 15 different sets of independent variables. The best performance is indicated in green. The predictors of IFN-γ, IL-5 and IL-4 are the best three predictors, indicating that the inexpensive cytokine can produce reliable predictions, whereas the predictor of IL-10 achieves poor performance. TNF and IL-6 appear among the predictors of all the models, indicating their strong associations with the expensive cytokines.

Independent Variables	$R^{2}$								
	IFN-γ			IL-10			IL-2		
	Linear	DT	RF	Linear	DT	RF	Linear	DT	RF
age	0.024	0.007	−0.202	−0.029	−0.200	−0.409	0.181	0.082	0.032
IL1β	−0.017	0.064	0.054	−0.030	−0.110	−0.123	−0.034	−0.127	−0.119
IL6	0.002	0.102	0.016	0.036	−0.016	−0.110	−0.032	0.111	0.094
TNF	0.201	0.454	0.377	−0.028	−0.185	−0.514	0.012	−0.034	−0.089
age + IL1β	0.026	−0.004	−0.055	−0.029	−0.272	−0.299	0.184	−0.077	−0.002
age + IL6	0.050	0.031	0.085	0.051	−0.071	0.013	0.196	0.111	0.230
age + TNF	0.216	0.506	0.566	−0.030	−0.236	−0.231	0.183	0.049	0.158
IL6 + IL1β	0.009	0.054	0.107	0.029	−0.143	−0.010	−0.039	0.177	0.180
TNF + IL1β	0.201	0.511	0.595	−0.037	−0.149	−0.163	−0.012	−0.176	−0.123
TNF + IL6	0.289	0.627	0.689	0.042	0.049	0.085	−0.035	0.060	0.081
age + IL6 + IL1β	0.072	−0.177	0.069	0.003	−0.361	0.005	0.161	−0.116	0.185
age + TNF + IL1β	0.258	0.575	0.710	−0.049	−0.377	−0.107	0.181	0.097	0.298
age + TNF + IL6	0.365	0.560	0.689	0.017	−0.189	0.089	0.207	0.141	0.357
TNF + IL6 + IL1β	0.335	0.670	0.743	0.028	−0.284	0.086	−0.027	−0.026	0.159
age + TNF + IL6 + IL1β	0.373	0.538	0.669	0.004	−0.356	0.126	0.185	0.076	0.347
**Independent Variables**	$R^{2}$								
	**IL-17A**			**IL-17F**					
	**Linear**	**DT**	**RF**	**Linear**	**DT**	**RF**			
age	−0.011	0.005	−0.155	0.065	−0.168	−0.268			
IL1β	−0.015	0.015	0.010	−0.019	−0.153	−0.135			
IL6	0.037	0.096	−0.036	−0.035	0.157	0.113			
TNF	−0.021	0.002	−0.066	0.007	−0.041	−0.159			
age + IL1β	−0.013	−0.043	−0.050	0.038	−0.306	−0.236			
age + IL6	0.056	−0.028	0.098	0.074	−0.365	−0.315			
age + TNF	0.024	−0.130	−0.044	0.104	−0.588	−0.093			
IL6 + IL1β	0.016	0.104	0.149	−0.024	−0.031	−0.014			
TNF + IL1β	0.029	0.071	−0.019	0.015	−0.126	−0.131			
TNF + IL6	0.104	0.153	0.145	0.031	−1.003	−0.399			
age + IL6 + IL1β	0.032	−0.023	0.206	0.012	−0.907	−0.347			
age + TNF + IL1β	0.036	−0.072	0.115	0.117	0.020	0.244			
age + TNF + IL6	0.134	−0.028	0.229	0.073	−0.027	0.263			
TNF + IL6 + IL1β	0.111	0.059	0.227	0.055	−0.931	−0.152			
age + TNF + IL6 + IL1β	0.110	0.045	0.299	0.122	−0.048	0.284			
**Independent Variables**	$R^{2}$								
	**IL-4**			**IL-5**					
	**Linear**	**DT**	**RF**	**Linear**	**DT**	**RF**			
age	0.004	−0.038	−0.143	0.107	0.101	−0.087			
IL1β	0.000	0.043	0.022	−0.026	−0.050	−0.056			
IL6	0.034	−0.020	−0.137	−0.031	0.088	0.072			
TNF	0.157	0.148	0.061	0.000	0.174	0.086			
age + IL1β	0.015	0.002	0.022	0.078	−0.051	0.017			
age + IL6	0.025	−0.164	−0.078	0.072	−0.031	0.176			
age + TNF	0.122	0.117	0.208	0.131	0.358	0.473			
IL6 + IL1β	0.047	−0.097	−0.066	−0.022	0.042	0.045			
TNF + IL1β	0.108	0.155	0.247	0.087	0.244	0.374			
TNF + IL6	0.124	0.287	0.463	0.037	0.607	0.648			
age + IL6 + IL1β	0.116	−0.197	0.089	0.093	−0.074	0.181			
age + TNF + IL1β	0.195	0.211	0.432	0.183	0.443	0.605			
age + TNF + IL6	0.150	0.286	0.476	0.131	0.561	0.690			
TNF + IL6 + IL1β	0.187	0.336	0.517	−0.017	0.540	0.658			
age + TNF + IL6 + IL1β	0.193	0.313	0.517	0.122	0.559	0.688			

**Table 11:** The number of occurrences of each of the input (inexpensive) cytokines, IL-6, TNF, IL-β and age in the top 10 ranked predictors for each of the expensive cytokines. Age is the most important feature for IL-2 and IL-17F. IL-β is the most important feature for IFNγ, IL-4 and IL-5, whereas IL-6 is the most important feature for IL-10 and IL-17A.

Target Cytokine	Input Cytokine			
	IL-6	TNF	IL-1β	Age
**IFN-γ**	6	10	6	5
**IL-10**	10	7	4	3
**IL-2**	7	6	5	10
**IL-17A**	9	8	6	5
**IL-17F**	7	7	4	8
**IL-4**	8	10	6	5
**IL-5**	8	10	5	6
